# A Traditional Clinic Chinese Medicine Prescription Qu-Zhuo-Tong-Bi (*QZTB*) Alleviates Gouty Arthritis in Model Rats

**DOI:** 10.1155/2019/9456318

**Published:** 2019-12-06

**Authors:** Huiqing Lv, Jianzhi Chen, Fenfen Liu, Yan Jin, Zhenghao Xu, Chengping Wen, Jie Yu

**Affiliations:** ^1^College of Pharmaceutical Science, Zhejiang Chinese Medical University, Hangzhou 310053, China; ^2^College of Basic Medical Science, Zhejiang Chinese Medical University, Hangzhou 310053, China; ^3^College of Stomatology, Zhejiang Chinese Medical University, Hangzhou 310053, China; ^4^Second Affiliated Hospital, Zhejiang Chinese Medical University, Hangzhou 310005, China

## Abstract

Qu-Zhuo-Tong-Bi (*QZTB*) is an empirical traditional Chinese medicine prescription for treating acute gouty arthritis clinically without serious adverse effects in mainland China. However, the biochemical mechanism underlying the therapeutic action produced by *QZTB* treatment against acute gouty arthritis and the effect on recurrent attack remain unknown. In this study, we investigated the anti-inflammatory and analgesic effects of *QZTB* on acute gouty arthritis and the recurrent attack in rats, as well as the underlying mechanisms. The gouty arthritis model was established by intra-articular injection of monosodium urate (MSU) crystal suspension (2 mg/50 *μ*L) into the right ankle joint of Sprague Dawley (SD) male rats. *QZTB* (500 mg/kg) and the positive control drug meloxicam were administrated by gavages twice a day for 7 days before, or 3 days after, first MSU injection in different experiments, respectively. The analgesic effects were evaluated by pain-like behaviors and hind paw mechanical withdrawal threshold testing. The anti-inflammatory activities were evaluated by ankle swelling measurement, histologic examination, NLRP3 inflammasome, and inflammatory cytokine expression. Western blot and quantitative real-time PCR were used to detect the protein and mRNA expressions of NLRP3. IL-1*β* and TNF-*α* level in the blood serum were detected by enzyme-linked immunosorbent assay (ELISA). *QZTB* can suppress ankle swelling and synovial inflammation in the MSU-induced gouty arthritis rat model. *QZTB* alleviated the acute attack and prevented the recurrent attack of gouty arthritis. In addition, *QZTB* treatment significantly decreased both mRNA and protein levels of NLRP3, as well as the production of IL-1 and TNF-*α* in the ankle joint of model rats. Taken together, these results suggest that *QZTB* may be a promising herbal formula for the prevention and treatment of gouty arthritis in humans.

## 1. Introduction

Gouty arthritis is an inflammatory disease caused by the deposition of monosodium urate (MSU) crystals in the joints, associated with purine metabolic disorder [1, 2]. Characterized by high serum uric acid level, acute inflammation, swelling of one or more synovial joints, and severe pain, it is commonly the first clinical manifestation of gout [3]. Recurrent attacks of gouty arthritis can lead to the formation of tophi and dense crystal deposits surrounded by fibrotic tissue, which cause disfigurement, bone destruction, and disability [4]. The management of gout, especially the recurrent acute attacks of chronic gouty arthritis, is still a problem to be resolved [5]. NLRP3 inflammasome, a member of nucleotide-binding oligomerization domain- (NOD-) like receptor (NLR) family, plays critical roles in gouty arthritis and many pathological inflammatory conditions [6, 7]. Current research studies show that MSU crystals trigger an inflammatory response through the activation of the NLRP3 inflammasome, which promotes IL-1*β* production. IL-1*β* can activate other proinflammatory cytokines, including tumor necrosis factor-*α* (TNF-*α*) which is critical for the initiation and propagation of the inflammatory response in gouty arthritis [8, 9]. Chemical compounds, such as nonsteroidal anti-inflammatory drugs, including naproxen and indomethacin, colchicine, corticosteroids, or a combination of them, are recommended as first-line systemic treatment for acute gouty arthritis [1, 10]. However, these drugs are associated with serious side effects, including chronic renal toxicity, gastrointestinal bleeding, and hepatic damage [11].

Traditional Chinese medicine (TCM) is an important part of complementary and alternative medicine, which has been widely applied to treat gouty arthritis and to cope with the complicated pathologic states of gout in different stages [12]. Our previous study has revealed that Qu-Zhuo-Tong-Bi (*QZTB*), an empirical traditional Chinese medicine prescription, has definite effects on treating hyperuricemia and gouty arthritis with less adverse reactions [13]. Furthermore, *QZTB* is a clinical experienced prescription, which has been widely prescribed to rheumatoid patients in China. Other functions of *QZTB*, such as reinforcing renal function, promoting blood circulation, and relieving pain, also have been clinically demonstrated [13–15]. However, the underlying mechanism of its anti-gouty arthritis and analgesic properties is still obscure.

Here, to investigate the acute anti-gouty arthritis and analgesic effect of *QZTB*, we applied the MSU crystal-induced animal model. And we further studied its effects on the recurrence of the second gouty attack. Our results demonstrated that *QZTB* had the potential of strong anti-inflammatory and analgesic effects and prevented the recurrent attack of gouty arthritis. The mechanism in anti-inflammation of *QZTB* could be contributed to the inhibition of the activation of the NLRP3 inflammasome.

## 2. Methods

### 2.1. Animals

Sprague Dawley male rats (180 ± 20 g body weight) were purchased from the experimental animal center of Zhejiang Chinese Medical University (SCXK (Yu)-2005-3001, Zhejiang Province, P.R. China). They were acclimatized for a week in a standard light- and temperature-controlled room at 22 ± 2°C, in 55 ± 10% relative humidity with a 12 h dark-light cycle, and they were freely fed with plentiful food and water. The animals were treated and cared for in accordance with the guidelines of experimental animal administration issued by the State Committee of Science and Technology of the People's Republic of China. The experimental protocol was approved by our departmental ethics committee.

### 2.2. Preparations of *QZTB* and Reagents

As reported in our previous papers [13, 16, 17], *QZTB* was composed of *Glabrous Greenbrier Rhizome* (Tu Fu Ling, 60 g), *Dioscorea septemloba Thunb.* (Bi Xie, 30 g), *Maydis stigma* (Yu Mi Xu, 15 g), *coix seed* (Mi Ren, 30 g), *Alismatis rhizome* (Ze Xie, 15 g), *Humulus scandens* (15 g), *Parasitc loranthus* (Sang Ji Sheng, 15 g), *Herba Siegesbeckiae* (Xi Qian Cao, 18 g), *Corydalis Rhizoma* (Yan Hu Suo, 18 g), *turmeric* (Jiang Huang, 12 g), and *Citrus medica* (12 g). All herbs were firstly soaked in 10 times distilled waters of their total weight for 1 h and then extracted twice with distilled water under reflux for 2 h. The filtered extracts were concentrated using a rotary evaporator at 50°C and freeze-dried into powder (*QZTB*). All the herbs were procured from Medical Pieces Co., Ltd., of Zhejiang Chinese Medical University (Hangzhou, Zhejiang, China). Meloxicam was purchased from Huadong Pharmaceuticals Ltd., Hangzhou, China. The *QZTB* powder and meloxicam were dissolved in 0.5% carboxymethyl cellulose in phosphate-buffered saline to the required concentration. Fresh solution was prepared before each experiment. The deionized water was purified by a Milli-Q system (Millipore, Bedford, USA). All other reagents used were of analytical grade and were purchased locally. The antibody against NLRP3 (cat# 15101) and *β*-actin (cat# 4970) was from Cell Signaling Technology (Danvers, MA). Levels of the cytokines IL-1*β* (cat# 583311, Cayman Chemicals Company, USA) and TNF-*α* (cat# 500850, Cayman Chemicals Company, USA) were measured by enzyme-linked immunosorbent assay (ELISA) kits according to the manufacturer's instructions. RNA isolation kit was from Qiagen (cat# 300112, Invitrogen, CA, USA).

### 2.3. Induction of Gouty Arthritis with MSU Crystals and Medicine Treatment

#### 2.3.1. Synthesis of MSU Crystals

The preparation of MSU crystals was reported previously [18–20]. Briefly, about 4 g of uric acid was dissolved and heated in 800 ml H_2_O with NaOH (9 ml/0.5 N), adjusted to pH 8.9 at 60°C, cooled overnight in a cold room, washed, and dried. Needle-like crystals were recovered and were suspended in sterile saline.

#### 2.3.2. MSU Crystal-Induced Gouty Arthritis in Animals

Adult male SD male rats were randomly divided into the following five groups, which consisted of 6–8 animals each: (1) the sham group (Sham), (2) gouty arthritis model group with MSU crystals injection (MSU + vehicle), (3) *QZTB*-treated group (500 mg/kg body weight) with MSU crystal injection (MSU + *QZTB*), and (4) the meloxicam-treated group (3 mg/kg body weight) with MSU crystal injection (MSU + meloxicam). 0.2 ml (4 mg) of MSU crystal suspension was injected into the right ankle joints [19, 21]. In order to evaluate the preventing efficacy, *QZTB* (500 mg/kg) and meloxicam (3 mg/kg) were orally administrated twice a day for 7 days before MSU injection. In the second experiment, *QZTB* (500 mg/kg) and meloxicam (3 mg/kg) were orally administrated for 3 days after the first injection of MSU crystal suspension. Following the treatment of *QZTB*, no evidence of systemic adverse effects was observed in any study group. The dosages of *QZTB* used in this study were based on those used in previous studies, and the dosages of meloxicam drugs were calculated based on the weight of rats [14, 15, 22]. *QZTB* and meloxicam were suspended in 0.5% carboxymethylcellulose in phosphate-buffered saline and administered.

### 2.4. Measurement of Ankle Swelling

The inflammation was quantified by measuring the ankle thickness (in millimeter) using a digital caliper at different time points for each rat before and 5 h, 24 h, 30 h, and 48 h after MSU crystal injections in the first experiment. The results were expressed as the test value of the ankle thickness. At the end of the experimental period (48 h) after the first or second MSU crystal injection, respectively, the rats were euthanized by isoflurane overdose. Blood samples were collected from the eye socket vein 24 h after MSU crystal injection and centrifuged at 3000 rpm for 10 min at 4°C, and the serum was collected and stored at −80°C until further tests.

### 2.5. Animals Were Assessed for Nociception (Touch Allodynia and Overt Pain-Like Behaviors) Post-MSU Injection

Measurements of touch allodynia (significant decrease in paw withdrawal threshold (PWT) compared with baseline values) were carried out by using von Frey monofilaments as previously reported [23]. Behavioral tests (*n* = 6–8 animals/group) were carried out 16 h after injection of MSU by blinded examiners. Animals were placed in a chamber with a mesh metal floor (20 × 30 cm), covered by an opaque plastic dome 10 cm high, and were always allowed to habituate for 1 h before any test. Withdrawal threshold to tactile stimulation was measured with a set of von Frey hairs with a bending force ranging from 2.0 to 26 g for the rats. Stimulation was applied to the plantar surface of the ipsilateral hind paw. Each hair was indented in the midplantar skin until it just bent. Clear paw withdrawal, shaking, or licking was considered as a nociception-like response. The filament of 8 g was used first. The stimulation was applied five times (several seconds for each trial) with an interval of at least 5 min. The strength of the next filament was decreased if the animal responded or increased if the animal did not respond. The minimum strength that evoked nociceptive responses at least three times out of the five trials was considered as the mechanical withdrawal threshold. Animals that did not respond to all filaments were given a maximal strength of 26.0 g. Overt pain-like behaviors induced by MSU injection were assessed using a standing scale from 0 to 3, as previously described by Coderre and Wall and dos Santos et al. [24, 25] (0: equal weight on both hind paws; 1: completely on the floor, but toes are not spread; 2: foot curled with only some parts of the foot lightly touching the floor; 3: foot elevated completely).

### 2.6. Histologic Examination

At the end of the experimental period (48 h) after first or second MSU crystal injection, respectively, the rats were deeply anesthetized using sodium pentobarbital (60 mg/kg, i.p.). The ankle joints of rats were isolated, fixed with neutral-buffered 10% formalin, and then decalcified with ethylenediaminetetraacetic acid (EDTA) for 3-4 weeks, as described previously [26]. Sections were cut at 12 *μ*m from the sagittal plane and then embedded in paraffin wax for histological analysis. The paraffin sections were stained with hematoxylin and eosin (H&E). The sections were evaluated via analyzing cell infiltration, pannus formation, and cartilage damage.

### 2.7. Measurement of Inflammatory Cytokines

IL-1*β*, a key cytokine in gout, is mediated by MSU crystals triggering the NLRP3 inflammasome [27]. To evaluate the anti-inflammatory effects of *QZTB* treatment in rats, we measured the inflammatory cytokines. Blood samples were incubated at room temperature for 30 min to clot and then centrifuged at 3000 rpm for 10 min at 4°C. The upper serum was transferred into a 1.5 ml microcentrifuge tube and stored at −80°C until further applications. The levels of TNF-*α* and IL-1*β* in serum were measured with enzyme-linked immunosorbent assay (ELISA) kits (Cayman Chemicals Company, USA) according to the manufacturer's protocol. The absorbance was read at 450 nm using a microplate reader.

### 2.8. Expression of NLRP3 Inflammasome in Ankle Joints

#### 2.8.1. Quantitative Real-Time PCR Analysis

Total RNA was isolated from treated ankle joints using TRIzol reagent (Invitrogen, CA, USA), and the concentration of RNA was determined using NanoDrop 2000 (Thermo Fisher Scientific, Wilmington, DE, USA). 0.5 *μ*g of RNAs was reverse-transcribed into cDNA with cDNA Reverse Transcription Kit (Bio-Rad, CA, USA). Quantitative PCR amplification was performed using a SYBR Green PCR master mix (Bio-Rad, CA, USA). Primers for rat NLRP3 and *β*-actin were synthesized by Sangon Biotech (Shanghai, China). The primer sequences are shown in Table 1. All real-time PCR experiments were run in quadruple. The mRNA expression levels of NLRP3 were analyzed using the ΔΔCt comparative quantification method following normalization to *β*-actin.

#### 2.8.2. Western Blot Analysis

The ankle joints were collected and ground into powders using liquid nitrogen. The joint powders were transferred into 1.5 mL Eppendorf (EP) tubes and lysed in cold lysis buffer containing 1 mM phenylmethylsulfonylfluoride (PMSF). The samples were then vortexed at high speed for 15 s, incubated on ice for 15 min, and vortexed again at high speed for 15 s. After centrifugation (15,000 g for 15 min at 4°C), the total proteins obtained in the supernatant were quantified using BCA protein assay kit (Tiangen Biotech Co., Ltd., China), according to the manufacturer's instructions. The proteins were mixed with a 5x loading buffer and heated at 100°C for 3 min to denature. Western blot was then performed using 10% SDS-PAGE. Proteins were transferred to PVDF membranes (Merk, Germany) (83 mm × 75 mm). After 1 h blocking with 5% dried skim milk dissolved in PBST (0.05% Tween 20), the membranes were individually incubated with primary antibodies overnight at 4°C and then incubated with secondary antibody for 1 h. The data were analyzed via densitometry using Molecular Analyst software (Bio-Rad Laboratories, Hercules, California, USA), and quantitated levels were normalized to their respective blotting from *β*-actin.

### 2.9. Statistical Analysis

The results were expressed as the mean ± sem and then analyzed by GraphPad Prism 8.0 (GraphPad Software, San Diego, CA, USA). One-way analysis of variance (ANOVA) was used followed by Dunnett's tests for multiple comparisons or unpaired Student's *t*-tests for two-group comparisons, and *P* values <0.05 were considered statistically significant.

## 3. Results

### 3.1. Effect of Pretreatment of *QZTB* on MSU Crystal-Induced Ankle Swelling

**Figure 1:**
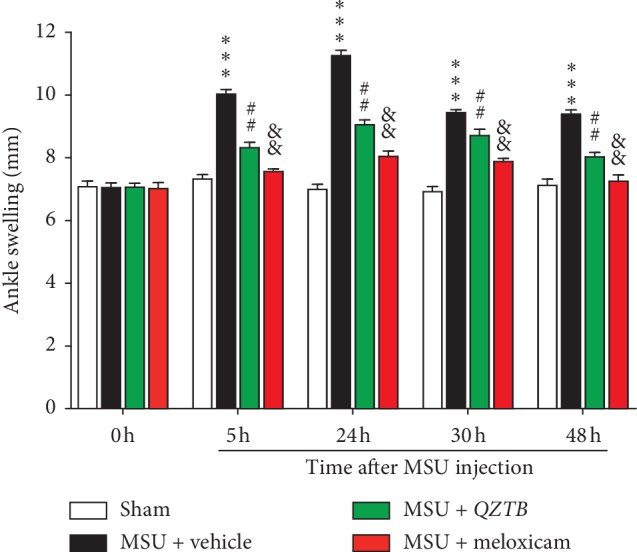
Effects of pretreatment of *QZTB* and meloxicam on MSU-induced gouty arthritis right ankle swelling in rats. *QZTB* (500 mg/kg·bw) and meloxicam (3 mg/kg·bw) decreased the ankle swelling. Values are expressed as mean ± sem of animals (*n* = 6–8). Comparisons were made as follows: ^*∗∗∗*^*P* < 0.001, MSU + vehicle vs. Sham; ^##^*P* < 0.01, MSU + *QZTB* vs. MSU + vehicle; ^&&^*P* < 0.01, MSU + meloxicam vs. MSU + vehicle. One-way analysis of variance (ANOVA) was used followed by Dunnett's tests.

To assess the effect of *QZTB* on the model of acute gout, we administered *QZTB* orally at 500 mg/kg twice a day for 7 days before MSU crystal injection (Figure 1). The dosage of *QZTB* is sufficient to reduce ankle swelling significantly in the preliminary experiment. On 0 h, 5 h, 24 h, 30 h, and 48 h after the first MSU crystal injection, we measured the ankle thickness of MSU crystal-induced rats. As shown in Figure 1, there was no difference in ankle diameters among all the experimental rats before model establishment. MSU crystals injection caused an increase in ankle diameter; the swelling reached a maximum at 24 h but gradually decreased at 30 h and 48 h, whereas *QZTB* (500 mg/kg) and meloxicam (3 mg/kg) treatment decreases the ankle swelling significantly. *QZTB* was able to inhibit the edema and presented inhibitory activity (*P* < 0.001) at 5 h, 24 h, 30 h, and 48 h after MSU injection with 24.5, 28.5, 16.5, and 22.7% of inhibition, respectively. Our study demonstrated that pretreatment of *QZTB* was able to prevent the ankle swelling produced by injection of MSU crystals in rats, which confirms its clinical use.

### 3.2. Pretreatment of *QZTB* Attenuated MSU Crystal-Induced Arthritis Pain-Like Behavior and Mechanical Allodynia

**Figure 2:**
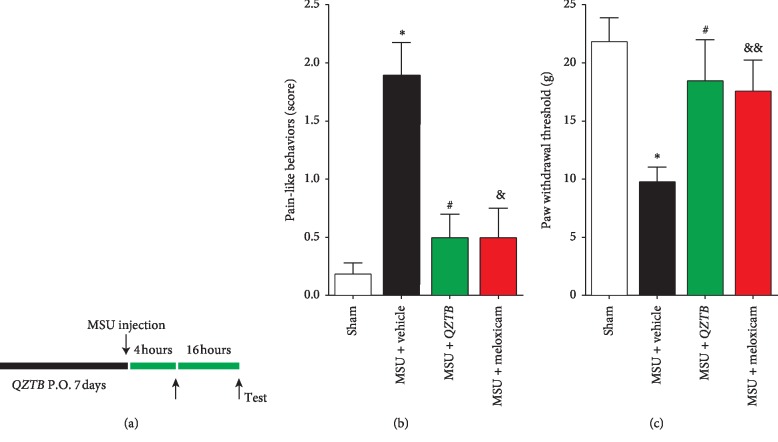
Effects of pretreatment of *QZTB* (500 mg/kg·bw) and meloxicam (3 mg/kg·bw) on pain-like behaviors and mechanical allodynia. Diagram of the experiment (a). *QZTB* (500 mg/kg.bw) and meloxicam (3 mg/kg·bw) decreased the pain-like behavior scores (b) and increased the paw withdraw thresholds (c) significantly. Comparisons were made as follows: ^*∗*^*P* < 0.05, MSU + vehicle vs. Sham; ^#^*P* < 0.05, MSU + *QZTB* vs. MSU + vehicle; ^&^*P* < 0.05, ^&&^*P* < 0.01, MSU + meloxicam vs. MSU + vehicle. One-way analysis of variance (ANOVA) was used followed by Dunnett's tests (*n* = 6–8).

To determine whether pretreatment of *QZTB* prevents arthritis pain-like behavior and the development of mechanical allodynia, pain-like behavior and allodynia tests were performed 4 hours or 16 hours after the first MSU injection, respectively. We found that the MSU injection resulted in an increase of pain-like behavior scores (*P* < 0.05) and prominent mechanical allodynia (*P* < 0.05), as shown in MSU-vehicle group in Figure 2. Pretreatment of *QZTB* and meloxicam decreased the pain-like behavior scores and increased the paw withdrawal thresholds, as shown in Figure 2.

### 3.3. *QZTB* Attenuated Second Injections of MSU Crystal-Induced Recurrence of Arthritis Pain-Like Behavior and Mechanical Allodynia

**Figure 3:**
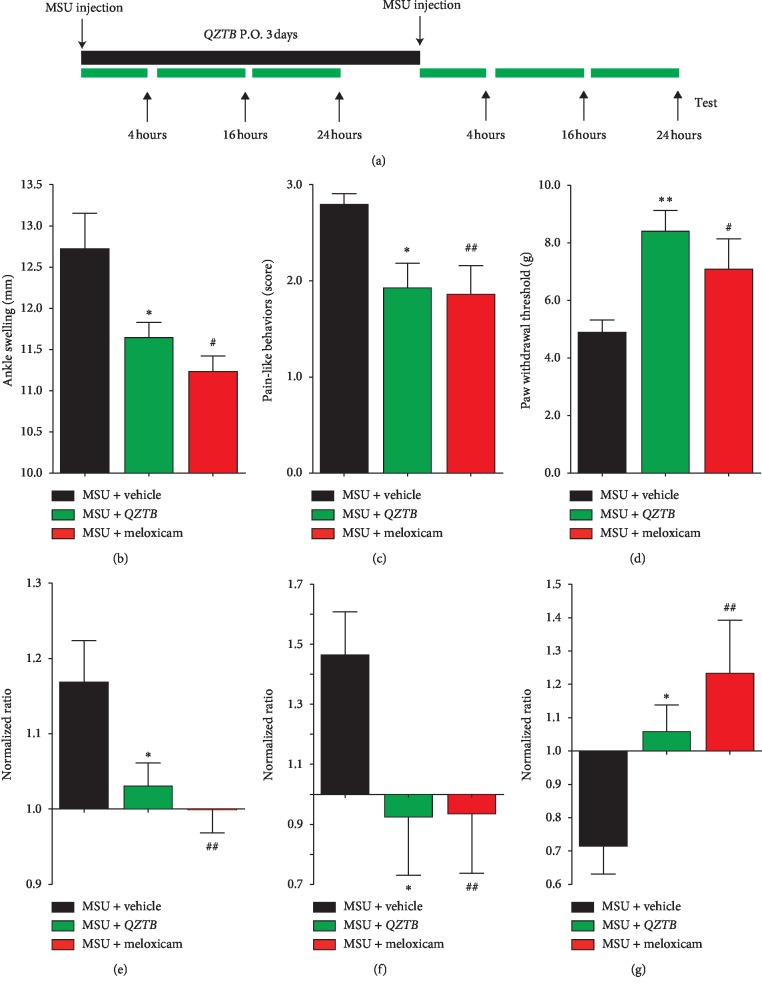
*QZTB* attenuated second injections of MSU crystal-induced recurrence of arthritis pain-like behavior and mechanical allodynia. Diagram of the experiment (a). Effects of pretreatment of *QZTB* (500 mg/kg·bw) and meloxicam (3 mg/kg·bw) on ankle swelling (b), pain-like behavior scores (c), and paw withdrawal thresholds (d) after the second injection of MSU crystal; the normalized inhibition ratio of *QZTB* and meloxicam (behavior scores after the second injection/first injection of MSU) (e–g). ^*∗*^*P* < 0.05, ^*∗∗*^*P* < 0.01, MSU + *QZTB* vs. MSU + vehicle; ^#^*P* < 0.05,^##^*P* < 0.01, MSU + meloxicam vs. MSU + vehicle. One-way analysis of variance (ANOVA) was used followed by Dunnett's tests (*n* = 6–8).

Recurrent attack of gouty arthritis is a prominent problem in clinic. To determine the effects on recurrent attacks, *QZTB* was administered for 3 days after the first injection of MSU crystals. Ankle thickness was measured 24 h after every MSU injection. The pain-like behavior and mechanical allodynia were tested 4 hours or 16 hours after every injection of MSU crystal, respectively (Figure 3(a)). We found that *QZTB* suppressed the joint edema (*P* < 0.05) and the pain-like behavior scores (*P* < 0.05) and increased the paw withdrawal thresholds (*P* < 0.01) after the second injection of MSU crystal, as shown in Figures 3(b)–3(d). The inhibition of *QZTB* was also compared and normalized to the first injection of MSU and is shown in Figures 3(e)–3(g).

### 3.4. Effect of *QZTB* on MSU Crystal-Induced Ankle Histological Manifestations

**Figure 4:**
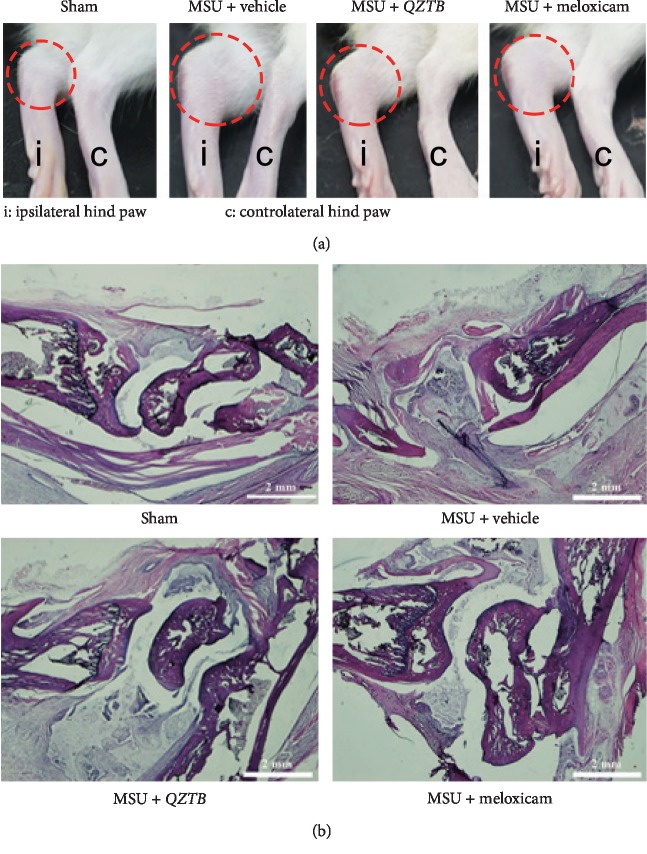
Effect of *QZTB* on MSU crystal-induced ankle histological manifestations. Photographs of hind paws of vehicle and experimental animals (a). Effects of pretreatment of *QZTB* (500 mg/kg·bw) and meloxicam (3 mg/kg·bw) on H&E-stained histological manifestations of the right ankle in rats 48 h after injection of 2.0 mg MSU crystal-induced gouty arthritis (b). Original magnification: ×20 (Sham: control rat; MSU + vehicle: MSU-induced rat; MSU + *QZTB*: *QZTB*-treated MSU-induced rat; MSU + meloxicam: meloxicam-treated MSU-induced rat.

To further assess anti-gouty arthritic activity of *QZTB*, joints synovial and surrounding tissues were removed from the ankle joint and analyzed using H&E staining. As shown in Figure 4, histological assay showed that there was no inflammation and infiltration of inflammatory cells in ankle joints control group. With a high-dose injection of MSU crystals (2 mg), gouty arthritis rats showed apparent joint inflammation, a number of inflammatory cells infiltration, and hyperplasia synovial. These pathological states were ameliorated to some degree by treating with *QZTB* and meloxicam. Although slight hyperplasia was observed in the synovial tissues of *QZTB* group, pretreatment with *QZTB* could suppress the infiltration of inflammatory cells and relieve the symptoms of MSU crystal-induced acute arthritis compared with the model group.

### 3.5. Effects of *QZTB* on Expressions of NLRP3 in Ankle Joints

NLRP3 inflammasome has been suggested as an important target for inflammatory disease control. To investigate whether *QZTB* regulates the expression of NLRP3, we detected both mRNA and protein levels of NLRP3 by qPCR and western blot, respectively.

**Figure 5:**
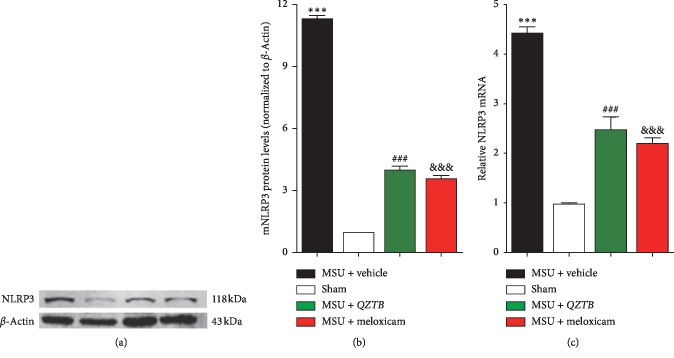
Effects of *QZTB* (500 mg/kg. bw) and meloxicam (3 mg/kg·bw) on mRNA and protein expressions of NLRP3 in ankle joints in MSU crystal-induced rats. Expression of NLRP3 protein and mRNA was determined by western blot analysis (a-b) and real-time PCR (c), respectively. The protein and mRNA expressions were normalized to *β*-actin. Values are expressed as mean ± sem. ^*∗∗∗*^*P* < 0.001, MSU + vehicle vs. Sham, ^###^*P* < 0.001 MSU + *QZTB* vs. MSU + vehicle, ^&&&^*P* < 0.001 MSU + meloxicam vs. MSU + vehicle. One-way analysis of variance (ANOVA) was used followed by Dunnett's tests (*n* = 3-4).

As shown in Figure 5, compared with the normal group, MSU crystals induced an upregulation of the NLRP3 expressions at both mRNA and protein levels in rat ankles (*P* < 0.001). *QZTB* could inhibit the mRNA (*P* < 0.001) and protein expression (*P* < 0.001) levels of NLRP3 compared to the MSU-induced model group. Meloxicam could also decrease the mRNA and protein expression levels of NLRP3 in ankle joints in MSU-induced gouty arthritis rats.

### 3.6. Effects of *QZTB* on Interleukin-1*β* (IL-1*β*) and Tumor Necrosis Factor-*α* (TNF-*α*) Production

**Figure 6:**
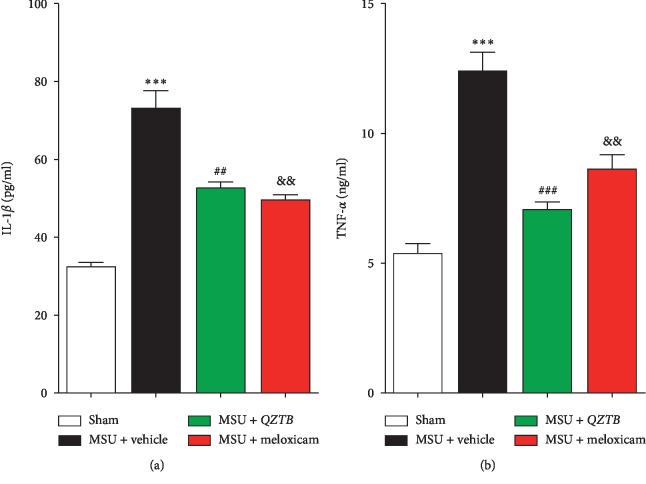
Effects of *QZTB* (500 mg/kg·bw) and meloxicam (3 mg/kg·bw) on IL-1*β* (a) and TNF-*α* (b) production in MSU crystal-induced rats. Values are expressed as mean ± sem (*n* = 3-4). The asterisks denote significance levels. ^*∗∗∗*^*P* < 0.001, MSU + vehicle vs. Sham; ^##^*P* < 0.01, ^###^*P* < 0.001, MSU + *QZTB* vs. MSU + vehicle; ^&&^*P* < 0.01 MSU + meloxicam vs. MSU + vehicle. One-way analysis of variance (ANOVA) was used followed by Dunnett's tests.

It has been reported that NLRP3 activation could lead to the increased release of IL-1*β* and TNF-*α*. As shown in Figure 6, elevation in serum levels of IL-1*β* (*P* < 0.001) and TNF-*α* (*P* < 0.001) was observed simultaneously in the MSU-induced rats. Compared to the MSU-induced group, meloxicam treatment (3 mg/kg) led to decrease in serum IL-1*β* (*P* < 0.01) and TNF-*α* (*P* < 0.01) level. *QZTB* suppressed the serum IL-1*β* (*P* < 0.01) and TNF-*α* (*P* < 0.001) levels (Figure 6) in the MSU crystal-induced rats, suggesting that *QZTB* can inhibit NLRP3 pathways contributing to arthritis in this acute gout model. These data suggested that *QZTB* can attenuate MSU-induced inflammation by suppressing the productions of IL-1*β* and TNF-*α* level.

## 4. Discussion

*QZTB* is an empirical traditional Chinese medicine prescription for clinical therapy of gouty arthritis. In the current study, the analgesic and anti-inflammatory effects of *QZTB* were examined in the MSU crystal-induced rats, and its potential mechanism was further investigated. Our results showed that *QZTB* could inhibit joint swelling, pain-like behavior, and allodynia caused by MSU crystal-induced inflammation, as well as joint inflammation, inflammatory cell infiltration, and hyperplasia synovial. The efficacy delivered by *QZTB* is comparable to meloxicam, which is the most frequently used drug for gouty arthritis clinical treatments. Moreover, *QZTB* could alleviate the recurrence of gouty arthritis, which is a prominent problem in clinic. The inhibition of NLRP3 by *QZTB*, evidenced by the decreased mRNA and protein expressions of NLRP3 in the ankle joint, and the lower production of serum IL-1*β* and TNF-*α* level, further indicates the efficacy of *QZTB* on gouty arthritis in clinic.

During acute gouty attacks, the affected joint exhibits the classic symptoms of inflammation, especially the intense pain in the affected joint. The acute gouty attack can cause severe pain that some patients even had the desire to amputate the affected limb [28]. Recurrent gouty arthritis attack remains a challenging problem. With the interval shortening over time, gouty arthritis, commonly known as the first clinical manifestation of gout, may progress to chronic arthritis and ultimately results in a decreased quality of life [3, 29]. Moreover, in the early period of urate-lowering therapy (ULT), it is always associated with acute and recurrent gouty attacks and the prevention of attacks is necessary [30, 31]. Therefore, the goals of gouty arthritis treatment are not only to decrease the serum uric acid level and terminate acute attacks but also to prevent recurrent gouty arthritis attacks and complications [32]. However, nonsteroidal anti-inflammatory drugs (NSAIDs), colchicine, or corticosteroids are usually recommended for a period of 6 months and even longer to prevent the recurrent [33]. Patients should be carefully monitored for adverse effects, especially for patients with renal dysfunction. Colchicine often causes gastrointestinal discomfort, kidney damage, bone marrow suppression, and other adverse reactions. NSAIDs can cause gastrointestinal bleeding, renal impairment, and cardiovascular events [11]. Long-term use of hormones can lead to endocrine metabolic disorders and digestive and cardiovascular complications. These side effects affected patient compliance to a certain extent; thus, the complementary and alternative medicines are desirable. Here, we found that *QZTB* could alleviate the pain-like behavior and the allodynia caused by intra-articular injection of MSU. Notably, *QZTB* prevented the swelling, pain-like behavior, and allodynia induced by the second injection of MSU, which means *QZTB* also could affect the recurrence of gouty arthritis.

Gouty arthritis is an inflammatory disease caused by deposition of MSU crystal in the joints as the serum uric acid level increases. Both uric acid and MSU released from tissue or cell damage can activate the NOD-like receptor family (pyrin domain containing 3, NLRP3). The NLRP3 inflammasome, a protein complex, has been reported playing an important role in the MSU-induced gouty arthritis [6, 34]. Here, we found that *QZTB* decreased both mRNA and protein levels of NLRP3, which may attribute to its effects on lowering uric acid as reported in our previous study [13]. Activated NLRP3 induces the conversion of procaspase-1 to active caspase-1, which in turn cleaves the inactive precursor cytokine pro-IL-1*β* into proinflammatory IL-1*β* [27]. The activation of IL-1*β* signaling pathway causes the transcription of proinflammatory cytokines such as TNF-*α*, which ultimately leads to inflammatory responses. Consistent with these results, we further demonstrated that *QZTB* decreases TNF-*α* and IL-1*β* levels, which greatly prove the alleviation of *QZTB* on pain and ankle swelling. Thus, combined with our previous study that *QZTB* exhibits efficacy in the treatment of hyperuricemia [13], it further suggests that *QZTB* would have high potential to treat the acute pain and recurrence of gouty arthritis in clinic.

The main problems of an empirical traditional Chinese medicine prescription for the treatment of health problems are chemical composition of the prescription and its quality control. In our previous reports, we identified four main components from *QZTB*, which are astilbin, diosgenin, curcumin, and quercetin, respectively. Although it is still unclear that which chemical components of *QZTB* contribute to its anti-inflammatory and anti-gouty arthritis effects in the present study. According to our previous studies [13, 16], astilbin could be the main functional composition of *QZTB*. The identification of the effective ingredients in *QZTB*, the related molecular biological mechanism, and the clinical research requires further in-depth investigation.

## 5. Conclusion

The present study demonstrated for the first time that *QZTB*, an empirical traditional Chinese medicine prescription in clinic, suppressed MSU crystal-induced swelling and pain in rats and exerted anti-inflammatory effects. The potential anti-gouty arthritis effect of *QZTB* may be attributed to the inhibition of NLRP3 inflammasome and downstream proinflammatory cytokines (IL-1*β* and TNF-*α*) levels. These findings indicate that *QZTB* may be a promising therapeutic formula for the prevention and treatment of gouty arthritis, especially of recurrent attacks, in a clinical setting.

## Figures and Tables

**Table 1:** Real-time PCR primer sequences.

Gene	Forward	Reverse
NLRP3	5′-GATGAACACTTGGAGCCCGT-3′	5′-GACTGGTGGGTTTGGGTCAG-3′
*β*-Actin	5′-ACAGGATGCAGAAGGAGATTAC-3′	5′-ACAGTGAGGCCAGGATAGA-3′

## Data Availability

The data used to support the findings of this study are available from the corresponding author upon request.

## Abbreviations

MSU: Monosodium urate
*QZTB*: Qu-Zhuo-Tong-Bi formula
IL-1*β*: Interleukin-1-beta
TNF-*α*: Tumor necrosis factor-alpha
NLRP3: NOD-like receptor family pyrin domain-containing 3
NSAIDs: Nonsteroidal anti-inflammatory drugs
TCM: Traditional Chinese medicine.
